# Provenance, paleoweathering, sediment maturity, and depositional environment of intertrappean sediments in Angot–Gazo volcanic plateau, northeastern Ethiopia: Evidence from field mapping, petrography and geochemistry

**DOI:** 10.1016/j.heliyon.2024.e38542

**Published:** 2024-09-26

**Authors:** Adise Zemelak, Worash Getaneh, Dereje Ayalew, Dejen Teka, Abebaw Bitew, Asaye Getenet

**Affiliations:** a Department of Geology, Woldia University, Woldia, Ethiopia; b School of Earth Science, Addis Ababa University, Addis Ababa, Ethiopia

**Keywords:** Ethiopian highlands, Intertrappean sediments, Arkosic graywacke, Provenance, Paleoweathering, Maturity

## Abstract

Numerous intertrappean beds have been reported in different sections of the Ethiopian highlands; however, their detailed paleo-sedimentological characteristics have not been fully examined. This study investigates the source rock composition, tectonic setting, degree of past weathering, paleoclimatic conditions, sediment maturity, and depositional environments of the Angot–Gazo terrestrial sediments through geological mapping, mineralogical analysis, and geochemical approaches. Two terrestrial beds, consisting of mudrock and sandstone, were identified. The sandstone is characterized by massive, medium to coarse, and poorly sorted grains that range from sub-rounded to angular shapes. Minerallogically, the sandstone comprises quartz, feldspar, and lithic fragments grains with a proportional amounts of ash material. Both mineralogical (Q–F–R) and geochemical classification plots categorize the sandstone as arkosic to lithic greywacke. Felsic volcanic rocks are the main source material for the investigated sediments, as evidenced by multiple discrimination plots (DF1 vs. DF2, V–Ni–Th∗10, La–Th–Sc, Cr/Th–Th/Sc, La/Co–Th/Co, La/Sc–Th/Co and Y/Ni–Cr/V), LREE/HREE patterns, elemental ratios, and the appearance of abundant felsic-derived lithic fragments within the sandstone. A new bivariate discriminant function plot from DF1 vs. DF2 analysis, coupled with ternary diagrams of Zr/10–Th–Sc for the studied intertrappean sediments, revealed a continental rift setting, particularly a passive continental margin. Ternary diagram of A–CN–K, binary plots of Al₂O₃/Na₂O vs. average values of weathering indices (CIA, CIW, PIA, & MIA), and SiO_2_ vs. Al_2_O_3_+K_2_O + Na_2_O plot demonstrate slight to moderate degree of weathering for the sandstone, and deep weathering for the mudrock under semi-arid paleoclimatic conditions. The binary schemes of SiO_2_/Al_2_O_3_ and Ni–TiO_2_ ratio, along with the significant abundance of angular to sub-angular framework grains, reflect both immaturity and initial sediment recycling. Finally, the examined sediments were deposited in freshwater under oxic conditions, as evidenced by binary plots of Sr/Ba, Th/U, U/Th, and Ni/Co.

## Highlights

• The Ethiopian highlands contain many intertrappean beds between volcanic rocks.
• The Angot–Gazo intertrappean beds consist of sandstone and mudrock, situated between felsic volcanic rocks.
• The sandstone samples are mineralogically, texturally, and chemically immature, and are classified as arkosic to lithic graywacke.
• The studied intertrappean sediments are primarily derived from felsic volcanic rock and are typically associated with passive continental margin tectonic settings.
• The source rock of the sandstone underwent slight to moderate weathering in a semi-arid paleoclimatic, ultimately being depositing in an oxygen-rich environment.

## 1 Introduction

**Fig. 1:**
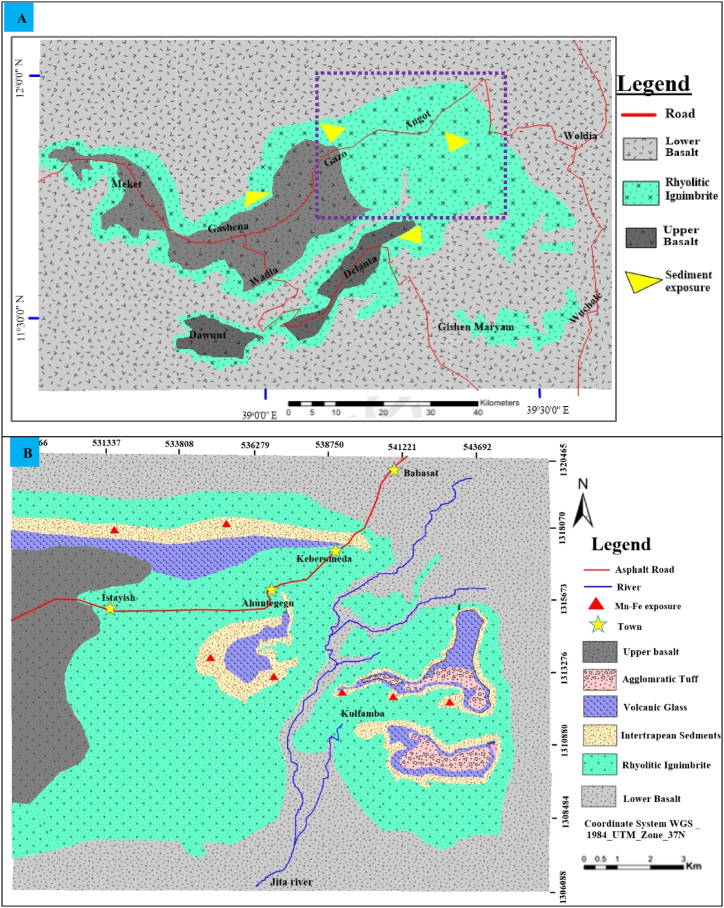
(A) Regional geological map of the Wollo volcanic plateau and the study area, highlighted by a violent dashed line (modified from Getaneh et al., 2022), and (B) detailed geological map of the Angot–Gazo area.

Intertrappean sediments were deposited during periods of volcanic quiescence, which lasted an average of 1–3 million years, interrupting the massive eruption of Oligocene volcanic rocks across Eastern Africa and Western Arabia [1,2]. In the Ethiopian plateaus, intercalations of terrestrial beds such as mudstones, siltstone, channelized sandstone, diatomites, lacustrine limestone, and coal seams with volcanic rocks have been reported [3]. According to the writers, these terrestrial beds are characterized by relatively limited thickness and lateral continuity compared to Mesozoic sedimentary rocks. The thickness varies from about 10 m to a few hundred meters in graben-related basins and their lateral continuity ranges from a few hundred meters to several kilometers. The stratigraphic position and age of the volcanic rocks containing the sediments can be used to determine the age of the terrestrial sediments. This is due to the fact that the Ethiopian intertrappean layers, which have been extensively studied by several scholars, are mostly enclosed between two basalts or between basalt and rhyolite. In the Ethiopian volcanic plateau, more than twenty-five intertrappean beds have been reported by scholars over the past five years, many of which contains coal seams and oil shale [1,4]. Among these, only the Wuchale and Magdala intertrappean sediments on the Wollo plateau have been slightly described by researchers. Coarse sandstones, silicified trunks, and whitish and black shale sediments alternating with volcanic rock were described in Magdala localities [5]. Additionally, the Ashangi Trap series' basaltic substratum in the Wuchale region has a coal-bearing sedimentary sequence [2]. It comprises two layers of coal, along with argillaceous sediment, carbonaceous material, and oil shale. Later, Ayalew et al. (2021) wrote about the existence of new terrestrial beds, it contains mudstone, sandstone, and conglomerate, representing approximately 3 million years episodes of terrestrial sedimentation in the northeastern Ethiopian plateau [6]. Their work focused on the stratigraphic outline of the northeastern Ethiopian highland, with detailed explanations of volcanic rocks based on field logging. The study develops the regional geological map of the northeastern Ethiopian highlands (Fig. 1A), and focusing solely on the stratigraphic positions of the terrestrial sediments, without providing detailed paleo-sedimentological descriptions. Accordingly, the present study is mainly concentrates on these slightly mentioned intertrappean sedimentary rocks.

In addition, all of the previously mentioned terrestrial beds were found in the lower and middle sections of Ethiopian plateau, either between two baslt flows or between basalt and rhyolite folws. In contrast, the current terrestrial sedimentary rock rests atop a thick sequence of bimodal continental flood products, sandwiched between felsic rocks. The sediments are underlain by rhyolitic ignimbrite and overlain by a volcanic glass units, and they host manganese–iron ore body. Consequently, the Angot–Gazo volcano–sedimentary basin represents the youngest terrestrial sediment in both northeastern volcanic suite and the entire Ethiopian volcanic plateau. However, aspects such as source rock composition, tectonic setting, paleo-weathering and climate, sediments maturity as well as paleo-redox conditions have not been fully studied. This study encompasses the geological and geochemical aspects of the Angot–Gazo intertrappean sedimentary rocks through comprehensive geological mapping, petrographic examination, and geochemical interpretation.

## 2 Regional geological setting

The breakage of the Afro-Arabian plateau and the development of the Afar rift are geodynamic phenomena associated with the formation of the Afar mantle plume [7]. This was followed by intense magmatic and tectonic activities throughout the region, including the Red Sea, Gulf of Aden, and Ethiopian Rift, during the lower Tertiary period. As a result, a huge amount of volcanic rocks covers a large part of the Ethiopian plateau. Cenozoic magmatic products in East Africa span at least 720,000 km^3^, with the Ethiopian continental flood basalt alone covering an area of 600,000 km [8].

The majority of the volcanic provinces in the Ethiopian highlands are bimodal in composition (basalt-rhyolite), with a noticeable absence of intermediate rocks [9]. In the northern Ethiopian Plateau, Cenozoic volcanism began with the formation of the Ashange basalt throughout the Eocene–Oligocene period, which was subsequently triggered by the eruption of Aiba basalts and Alajae rhyolite in the Oligocene–Miocene period [10]. After a significant amount of flood basalt emplacement, several large shield volcanoes, including Mt. Guna (10-7 Ma), Mt. Choke, and Gugugftu (22 Ma), were developed [11]. After the end of the Oligocene, volcanism migrated into the Afar depression due to the gradual stretching and shifting between the Somali plateau and the southern Ethiopian plateau, coinciding with the creation of the Main Ethiopian Rift [3].

In addition, many terrestrial beds have been reported among the volcanic rocks of Ethiopia [3,12]. The northeastern Ethiopian plateau, in particular, has been noted for containing young terrestrial sediments [6,13]. These sediments rest atop a 1600-m-thick layer of flood basalt and a 200-m-thick layer of felsic volcanic rocks. Unlike the previously mentioned intertrappean beds, the present terrestrial sediments are underlain by pyroclastic rocks such as ignimbrite, and are overlain by volcanic glass.

## 3 Sampling and methods

The Angot–Gazo intertrappean beds were investigated through fieldwork, petrographic analysis, and geochemical examinations. The research began with geological mapping, lithological descriptions, and the collection of representative samples. Rock unit thicknesses were measured using a handheld GPS (global positioning system) device by recording the top and bottom elevations and calculating the difference between them. Outcropped terrestrial beds were sampled in Istayish, Ahuntegegn, Keberomeda, Kulfamba, and Sinora slasie area for laboratory analysis. Ten thin sections (0.3 mm thick) were prepared at the Geological Survey of Ethiopia (GSE). The preparation involved removing weathered rock surfaces and cutting to a suitable size using diamond blades. Then, the textural descriptions, mineralogical identification, and modal proportions of the sandstone were determined using visual estimation under a transmitted light microscope.

In addition to the petrographic analysis, fifteen samples (ten sandstone and five mudrock) were analyzed for geochemical studies at the Australia Laboratory Service (ALS) in Ireland. The sample preparation procedure involved drying fresh samples in ovens at over 100 °C, crushing them into fine chips (70 % passing 2 mm), and pulverizing them into a fine powder (85 % passing 75 μm). This powder was analyzed for major, trace, and rare earth elements using a combination of Inductively Coupled Plasma-Atomic Emission Spectrum (ICP-AES) and Inductively Coupled Plasma-Mass Spectrometry (ICP-MS) via lithium metaborate fusion (FUS-LI01). A 0.200 g sample was mixed with 0.90 g of lithium metaborate flux, fused in a furnace at 1000 °C, and dissolved in 4 % HNO3/2 % HCl solution for major oxide analysis. Similarly, trace and rare earth elements were analyzed using the same fusion and dissolution process. The resulting solution was then analyzed by ICP-AES for major oxides and by ICP-MS for trace and rare earth elements. Loss On Ignition (LOI) was determined by heating a 1.0 g sample at 1000 °C for 1 h and calculating the weight loss. In this work, various provenance, tectonic setting, paleo-weathering index, paleoclimate, depositional environment, and sediment maturity discrimination diagrams were utilized by applying the formulas discussed in each sections.

## 4 Results

### 4.1 Field study

The Angot–Gazo plateau is distinguished by extensive outcrops of volcanic rocks, along with some terrestrial sedimentary rocks. Since the terrestrial sediments are sandwiched between volcanic rocks, it is preferable to mention them from oldest to youngest. The major lithological units include lower basalt (both porphyritic and aphanitic texture), rhyolitic ignimbrite, terrestrial sediments, volcanic glass, agglomeratic tuff, and upper basalt (characterized by calumniated aphanitic texture). These volcanic rocks and intertrappean sediments were mapped at a scale of 1:50,000, based on their lateral contact relationship (Fig. 1B). The geological map of the study area was produced through field surveys, supplemented by tools such as Google Earth and ArcGIS. The Angot–Gazo terrestrial beds (mudrock and sandstone) are sandwiched by ignimbrite on the bottom side and volcanic glass on the top. However, in some exposure, particularly in Istayish section, the rhyolite is underlined by terrestrial sediments. These sediments host ferromanganese metallic resources.

**Fig. 2:**
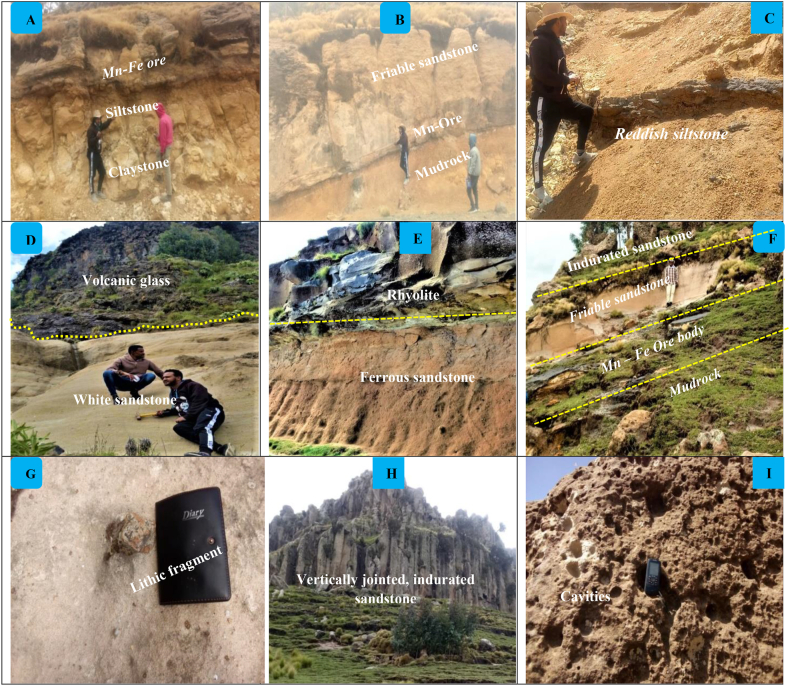
Intertrappean sediments under exposure level: (A–C) mudrock overlain by Mn–Fe ore-hosted sandstone, (D–G) friable sandstone, and (H–I) indurated sandstone in the study area.

In most parts of the plateau, mudrock crops out above the ignimbrite unit and below the ore body (Fig. 2B). In some localities like Istayish, this rock is found above the sandstone unit. In most exposures, a white, significantly altered pyroclastic tuff, up to 1 m thick marks the boundary between the underlying ignimbrite and mudrock. The rock is composed of clay- to silt-sized sediments and has a fragile nature with a clear mud crack structure. At different exposures, the thickness varies from 1 to 5 m. The color and size of the grains observed in the field helps to classify this rock as whitish, black, and reddish mudrock. The first type of mudrock is called claystone; it has a whitish color, massive structure, extremely fine, smooth, and soft materials, with a potential thickness of up to 2 m (Fig. 2A). This claystone cropped out on the eastern sides of the plateau, particularly in Kulfamba and Keberomeda localities. The second type is distinguished by its dark gray to black color, clay- to silt-sized, and moderately fissile, with a thickness reaching up to 1.5 m. It crops out in the uppermost parts of the sandstone, particularly in the Istayish section. The final mudrock has a reddish-brown color, silt-sized, and a rough texture, and is found throughout the plateau (Fig. 2A–C & F). It is relatively more dominant over the others, with thicknesses varying from a few centimeters to 5 m in different exposures.

Sandstone is found in the entire parts of the plateau, where it overlays mudrock and is primarily underlain by volcanic glass, and to some extent by rhyolite (Fig. 2D and E). Based on the degree of compacting and structural features observed on the field, the sandstone can be classified into two types: indurated and friable sandstone. The former is poorly compacted, friable, and has a light gray to reddish-brown color with thicknesses ranging from 8 to 30 m in Keberomed and Istayish sections respectively (Fig. 2B, D–F). Some pebble- to cobble-sized rock fragments, ranging from a few millimeters to 10 cm in diameter, are incorporated and associated with coarse-grained quartz and feldspar minerals (Fig. 2G). The second type of sandstone is well-indurated, has a whitish to light gray color, and its thickness varies from 30 to 40 m in the Angot district, particularly in the Kulfamba locality. However, it is not found in the Istayish and Ahuntegegn sections. This indurated sandstone is distinguished by the unusual occurrence of large sandstone boulders and a vertical jointing system, with joint spacing and aperture reaching 60 and 40 cm, respectively (Fig. 2F, H & I). Another characteristic that distinguishes it from the friable sandstone is the existence of tiny to big holes (Fig. 2I). The influence of surficial weathering is responsible for these holes. The former sandstone is the main host rock for layered Mn–Fe ore, while the latter one hosts sporadic, chemically precipitated manganese coating and non-systematically oriented Mn–Fe veins. In general, the thickness of the terrestrial sediment varies from 8 to 55 m. This thickness variation is a result of paleomorphological influences that occurred during the sedimentation process.

### 4.2 Petrography

**Fig. 3:**
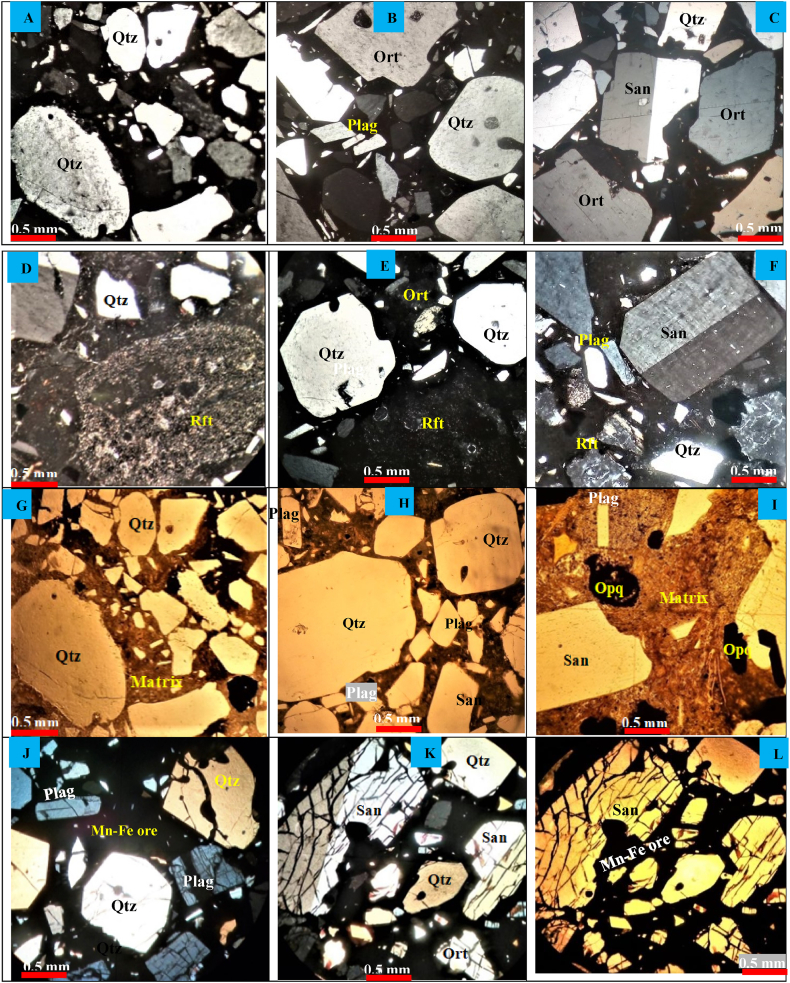
Petrographic observation of Angot–Gazo sandstone under XPL (cross polarizing light) and PPL (plane polarizing light) reveal the following: (A–F) XPL views show the sandstone comprises angular to sub-rounded quartz (Qtz), sanidine (San), orthoclase (Ort), plagioclase (Plag), rock fragments (Rft), and volcanic ash as a matrix; (G–I) PPL views display the same mineral grains along with additional opaque minerals (Opq); and (J–L) the mineral-bearing sandstone contains Mn-Fe ore (seen in all the dark regions), along with quartz, sanidine, orthoclase, and plagioclase feldspar. The final three images illustrate extensive dissection of framework grains, infiltrated and filled by later-coming Mn-Fe-rich fluids.

The Angot–Gazo intertrappean sandstone is composed of quartz, feldspar, lithic fragment, ash material as groundmass, and a few opaque minerals (Fig. 3A–L). On average, the sandstone is consists of 30 % quartz, 28 % feldspar, 27 % matrix, 15 % lithic fragments, and a small amount of opaque minerals (Table 1). The quartz grains are predominantly monocrystalline (occur as single grain), sub-rounded to angular, slightly fractured, and exhibit white transparent color with a hexagonal crystal habit. Additionally, it shows undulatory extinction, which illustrates the sweeping of extinction during stage rotation. The feldspar, particularly sanidine minerals, exhibit Carlsbad twining, and most crystals have a flattened or tabular habit (Fig. 3C, F, K, and L). In addition, there are untwinned feldspar crystals with tabular and irregular shapes (Fig. 3B, C, & J–L), likely orthoclase and plagioclase. Even though these feldspars are untwinned, they display one set of cleavage. This is a key feature for distinguishing feldspar from quartz, as quartz does not show cleavage. Similarly, the lithic fragments are clearly observed under petrographic view (Fig. 3D, E, & F). The rock fragments display a range of grain sizes, exhibit a dark gray to light brown color under XPL (cross polarizing light) and PPL (plane polarizing light) views. They are composed of feldspar, glassy materials, quartz, and ash materials. The opaque minerals have been also observed associated with those framework grains, and they have dark black color under PPL views (Fig. 3G–I). Furthermore, Mn–Fe hosted sandstone was examined under a transmitted light microscope (Fig. 3J–L). This sandstone consists of manganese-iron oxide/hydroxide minerals, along with quartz and feldspar. Not only feldspar but also quartz minerals are significantly affected and dissected by later-emplaced ferromanganese-rich hydrothermal fluids. As a result, the fractured grains are filled by Mn–Fe ore minerals, forming micro-veins.

**Table 1:** Recalculated framework grains and matrix of Angot–Gazo intertrappean sandstone. The last three columns show recalculated Q-F-R values, expressed as percentages by removing the matrix.

Sample No	Quartz	Feldspar	Rock fragment	Matrix	Q (100 %)	F (100 %)	R (100 %)
An −1	25	30	10	35	38.46	46.15	15.38
An −2	35	35	5	25	46.66	46.66	6.66
An −3	15	20	30	35	23.08	30.77	46.15
An −4	45	20	5	30	64.28	28.57	7.14
An −5	20	40	10	30	28.57	57.14	14.28
An −6	40	15	20	25	53.33	20	26.66
Ga −1	35	10	35	20	43.75	12.5	43.75
Ga −2	20	45	5	30	28.57	64.28	7.14
Ga −3	50	20	5	25	66.66	26.66	6.66
Ga −4	35	45	0	20	43.75	56.25	0
Average	30	28	15	27	41.10	38.36	20.55

### 4.3 Geochemistry

To verify the microscopic and megascopic investigations in this study, geochemical analysis offers a significant advantage. Therefore, major and trace elements along with REE patterns, were utilized to identify the tectonic setting, paleoweathering-climate, sediment maturity and source rock from which the terrestrial deposits originated. Table 2, Table 3 provide the major, trace, and REEs compositions of the examined sediments, while Table 4 summarizes the element ratios. The examined sandstone shows high concentrations of SiO_2_ (avg. 71.9 wt%), Al_2_O_3_ (avg. 12.8 wt%), Fe_2_O_3_(avg. 4.24 wt%), K_2_O (avg. 3.7 wt%), and Na_2_O (avg. 3.2 wt%). The contents of the first three major oxides contrast from 66.8 to 78.9 wt%, 10.4–15.3 wt%, and 2.38–8.4 wt%, respectively, while the latter two oxides exhibits consistent levels across the samples. The remaining oxide components show a little bit variation between samples and have low concentrations (less than 1 %). The sandstone samples exhibited comparatively higher iron oxide content, indicating that the opaque minerals identified in the thin-sections are ferrous minerals. The average Na₂O content of 3.2 wt% in the investigated sandstone suggests the presence of plagioclase feldspar. However, potassium feldspar is present in substantially greater concentrations than plagioclase feldspar, as evidenced by the relative enrichment of K_2_O (avg. 3.7 wt%) compared to Na_2_O (avg. 3.2 wt%) in the Angot–Gazo sediments. The K_2_O/Na_2_O ratios vary from 0.86 to 1.36, with an average of 1.16 wt%. These concentration and ratio results align with petrographic examination, which indicates that K-feldspar predominates over other feldspar minerals. In addition, the trace element content of the studied sediments shows notable enrichment in high field strength elements, for example Zr, Hf, Y, Ta, Nb, Sm, and rare earth elements (La, Ce, Nd, Tm, Yb) in comparison to the upper continental crust (UCC). In contrast, Co, Sc, Ni, Sr, and Cr are highly depleted, with moderate depletion observed in U, Th, Ce, Rb, and Ba relative to the UCC (Table 4).

**Table 2:** Major oxide compositions (wt. %) and calculated parameters of Angot–Gazo intertrappean sedimentary rocks (mudrock and sandstone).

Sample code	An 01	An 02	An 03	An 04	Ga 01	An 05	An 06	An 07	An 08	An 09	An 10	Ga 02	Ga 03	Ga 04	Ga 05
SiO_2_	45	45.3	48.8	48.5	46.6	77.5	73.9	78.93	77	66.8	67	67.2	67.5	76.1	67
SiO_2_adj	54.88	53.91	56.79	55.91	56.17	78.31	74.69	83.47	77.18	70.59	69.98	70.22	71.04	76.57	70.97
Al_2_O_3_	24.9	26.3	25.7	27.4	12.05	10.7	12.4	10.44	11.4	14.45	13.65	15.3	14.25	12.9	13.45
Fe_2_O_3_	9.26	8.02	7.28	8.8	10.25	2.45	3.01	2.38	2.68	5.13	8.41	4.82	4.48	2.56	6.53
CaO	0.69	0.4	0.31	0.22	4.54	0.15	0.1	0.13	0.12	0.49	0.11	0.58	0.27	0.11	0.39
CaO∗	0.13	0.4	0.31	0.09	1.04	0.15	0.1	0.13	0.12	0.49	0.11	0.58	0.27	0.11	0.39
Na_2_O	0.13	0.87	0.86	0.09	1.04	3.2	3.87	3.17	3.31	3.03	2.7	2.79	3.41	3.36	3.21
K_2_O	0.18	0.83	0.82	0.13	0.89	4.33	4.91	3.75	4.53	3.34	3.04	3.29	3.72	3.66	2.77
MgO	0.39	0.23	0.16	0.08	3.75	0.07	0.06	0.08	0.09	0.29	0.04	0.44	0.22	0.05	0.21
TiO_2_	1.35	1.9	1.86	1.45	2.89	0.45	0.55	0.44	0.49	0.91	0.62	1	0.93	0.51	0.7
MnO	0.04	0.09	0.06	0.02	0.37	0.06	0.1	0.06	0.09	0.06	0.09	0.17	0.17	0.09	0.06
P_2_O_5_	0.02	0.06	0.05	0.03	0.48	0.03	0.02	0.02	0.02	0.05	0.05	0.07	0.02	0.03	0.04
BaO	0.02	0.02	0.02	0.01	0.07	0.02	0.02	0.01	0.02	0.06	0.02	0.03	0.04	0.02	0.05
LOI	18.2	14.25	13.05	13.65	18.1	1.8	1.19	2.24	1.54	5.72	4.42	5.96	4.02	2.43	5.23
Total	100.19	98.28	98.98	100.39	101.06	100.76	100.13	96.8	101.3	100.34	100.15	101.66	99.03	101.82	99.64
CIA	98.26	92.60	92.81	98.88	80.22	58.21	58.27	59.69	58.88	67.80	70	69.67	65.81	64.40	67.86
CIW	98.96	95.39	95.64	99.34	85.27	76.15	75.74	75.98	76.87	80.41	82.92	81.94	79.47	78.80	78.88
PIA	98.96	95.25	95.51	99.34	84.29	65.53	65.35	66.96	66.69	75.94	79.06	78.08	74.10	72.69	74.789
MIA	96.52	85.21	85.62	97.76	60.45	16.43	16.54	19.38	17.76	35.61	40	39.34	31.64	28.80	35.72
Avg.value of CIA, CIW, PIA, MIA	98.18	92.1	92.39	98.83	77.56	54.08	53.98	55.5	55.05	64.94	67.99	67.26	62.76	61.17	64.31
SiO_2_/Al_2_O_3_	2.20	2.05	2.21	2.04	4.66	7.32	6.02	7.99	6.77	5.06	5.13	4.85	5.12	5.94	5.27
Log (SiO_2_/Al_2_O_3_)	0.26	0.24	0.28	0.25	0.59	0.86	0.77	0.88	0.83	0.66	0.69	0.64	0.67	0.77	0.69
Log (Fe_2_O_3_/K_2_O)	1.71	0.98	0.95	1.83	1.06	−0.25	−0.21	−0.19	−0.23	0.18	0.44	0.16	0.08	−0.15	0.37
Log (Na_2_O/K_2_O)	−0.14	0.020	0.021	−0.16	0.07	−0.13	−0.10	−0.07	−0.13	−0.04	−0.05	−0.07	−0.04	−0.04	0.06
Log (K_2_O/Na_2_O)	−1.11	0.22	1.59	0.09	−0.18	0.01	0.22	0.01	0.69	0.478	0.478	0.60	0.30	0.48	0.42
DF1 (Arc-rift-Col) Hs	−1.08	−1.54	−1.38	−1.32	0.16	−6.68	−7.17	−6.24	−5.95	−4.79	−9.72	−3.05	−4.42	−7.27	−5.93
DF2 (Arc-rift-Col) Hs	−0.99	0.36	0.99	1.01	−1.48	−19.3	−18.7	−19.2	−18.7	−15.5	−17.9	−14.6	−15.5	−18.7	−15.8

**Table 3:** Trace element compositions (ppm) of analyzed Angot–Gazo intertrappean sedimentary rocks (mudrock and sandstone).

Sample code	An 01	An 02	An 03	An 04	Ga 01	An 05	An 06	An 07	An 08	An 09	An 10	Ga 02	Ga 03	Ga 04	Ga 05
Ba	159	205	188	124.5	593	177.5	176.5	174	480	163.5	252	188	336	205	436
Cr	10	30	20	10	10	10	10	20	40	10	20	20	20	10	20
Cs	1.14	1.15	1.48	1.04	2.02	0.35	0.57	0.52	0.74	0.71	1.2	0.8	1.02	0.71	0.71
Ga	67.9	75.7	76.4	81.8	24.9	27.1	33.4	28.4	36.1	33	37.6	25.9	45.6	37.5	42
Hf	53	55.7	54	48.8	6	7.7	15.4	9.5	14.5	13.7	15.6	6.1	13.3	8.6	15
Nb	302	284	293	273	26.8	47.3	79.6	53	77.8	76.2	82.4	37	77.8	44.1	77.2
Rb	8.1	11.8	14.6	9.2	56.9	51.3	81.7	51.1	42.3	21.4	34.6	35.4	26.6	24.6	24.4
Sn	15	17	16	14	2	3	5	3	5	4	5	4	4	2	4
Sr	42.8	29.6	25.8	12.8	315	12.9	7	8.2	61.5	7.5	44.1	13.1	20.6	8.6	30.7
Ta	15.9	16.1	16	13.8	1.5	6.3	5.3	2.9	5.1	4.4	5.1	1.8	4.3	2.6	5.1
Th	35.4	36.5	36.1	30.5	3.6	5.57	10.9	6.63	12.1	9.96	10.6	4.5	11.55	5.79	9.78
U	6.59	6.55	9.87	3.88	1.01	1.64	2.89	1.62	2.54	1.6	2.62	1.3	1.47	1	2.95
V	67	91	77	31	308	12	16	15	38	27	48	32	17	12	45
W	4	6	7	4	<1	2	2	2	2	3	2	1	<1	<1	2
Zr	2080	2190	2200	2050	240	301	617	373	572	557	601	256	553	336	612
Ag	4.4	4.3	3.6	4.2	<0.5	0.6	1	0.8	0.9	1.3	0.7	0.8	<0.5	<0.5	<0.5
As	<5	8	11	8	<5	<5	<5	5	<5	<5	<5	5	<5	<5	5
Cd	<0.5	0.8	0.7	<0.5	0.5	<0.5	<0.5	<0.5	<0.5	<0.5	<0.5	0.5	<0.5	<0.5	<0.5
Co	1	4	1	1	26	1	1	1	4	2	6	1	1	1	2
Cu	<1	<1	<1	<1	18	1	<1	4	8	<1	2	1	2	2	4
Li	40	60	50	60	10	10	10	10	10	10	10	10	10	10	20
Mo	3	4	3	<1	1	1	1	<1	1	2	2	2	2	<1	2
Ni	4	11	3	2	10	5	6	5	12	4	5	5	4	4	5
Pb	47	52	51	39	4	9	12	9	13	10	15	3	9	5	13
Sc	6	8	8	11	21	4	5	4	7	6	5	4	4	4	11
Tl	<10	<10	<10	<10	<10	<10	<10	<10	<10	<10	<10	10	<10	<10	<10
Zn	217	235	210	103	127	50	128	91	122	79	156	57	98	46	99
La	111	194	344	47.8	32.8	99.2	46.2	46	150	84.2	118	77.6	95.2	119	80.7
Ce	82.7	420	448	38.8	69.2	202	105.5	138	239	98.3	236	161	108	131.5	219
Pr	28.7	48.2	85.5	13.9	9.53	25.1	14.2	14.3	34.8	22.6	31.7	19.8	24.1	27.7	20.8
Nd	115.5	165	292	53.2	42.7	97.8	54.9	54.7	134.5	81	124	77.5	89.9	106	84.1
Sm	25.2	28.4	47.5	12.9	9.82	15.95	11.65	10.3	24.1	14.2	22.2	12.7	17.5	18.5	18.45
Eu	5.72	5.71	9.21	3.69	3.23	3.05	2.68	2.23	5.22	3.05	4.77	2.49	4.12	4.03	4.75
Gd	22.7	20.1	32.6	14.25	9.95	10.55	10.05	7.67	19.55	9.76	17.5	8.76	15.75	15.35	15.8
Tb	3.84	3.59	4.92	3.5	1.27	1.44	1.73	1.21	2.81	1.41	2.6	1.21	2.22	1.96	2.21
Dy	24.2	23.8	30.8	28.5	7.3	7.57	10.85	7.04	15.15	7.96	14.7	6.09	13.75	11.55	11.95
Ho	5.03	5.39	6.51	6.77	1.3	1.41	2.23	1.3	2.78	1.51	2.94	1.1	2.65	1.99	1.97
Er	15.2	17.1	19.95	22.5	3.74	3.64	6.42	3.71	7.55	4.3	8.17	3.21	7.86	5.63	5.06
Tm	2.28	2.74	3.09	3.72	0.46	0.5	0.92	0.51	0.96	0.68	1.17	6.2	1.17	0.68	0.64
Yb	15.25	18	19.7	25.8	2.94	3.01	5.69	3.48	5.87	5.1	7.34	2.77	7.93	4.25	3.73
Lu	2.35	2.68	3.05	4.04	0.43	0.46	0.85	0.49	0.86	0.8	1.1	0.43	1.13	0.65	0.56
Y	113	140.5	169	165.5	35.6	33	56.1	31.7	70.2	34.7	77	60	69.4	70.1	47.9

Mudrock (An 01 – An 04 and Ga 01), Indurated sandstone (An 05 – An 08), Friable sandstone (An 09 – An 10 and Ga 02 – Ga 05).

**Table 4:** Range of elemental ratios for Angot–Gazo intertrappean sediments compared to felsic rocks, mafic rocks and the upper continental crust (UCC).

Lithotype	Angot–Gazo Intertrappean Mudrock						Angot–Gazo Intertrappean Sandstone											Ranges for felsic, mafic rocks and UCC		
Ratio	An- 14	An- 15	An- 16	An- 17	Ga-04	Avg.	An- 2	An- 3	An- 4	An- 5	An- 6	An- 8	Ga- 3	Ga- 4	Ga- 5	Ga- 6	Avg.	Felsic rocks	Mafic rocks	UCC
Al/Ti	18.4	13.8	13.8	18.9	4.2	**13.8**	23.7	22.5	23.7	23.3	15.9	22.0	15.3	15.3	25.3	19.2	**20.6**	21–70	3.00–8.00	30.34
La/Sc	18.5	24.3	43	4.34	1.56	**18.3**	24.8	9.24	11.5	21.5	14.0	23.6	19.4	23.8	29.8	7.34	**18.5**	2.50–16.3	0.43–0.86	2.21
La/Co	111	48.6	430	47.8	1.26	**127.7**	99.2	51.3	46	37.6	42.1	19.7	77.6	95.2	198	40.4	**70.7**	1.80–13.8	0.14–0.38	1.76
Th/Sc	5.9	4.56	4.51	2.77	0.17	**3.58**	1.39	2.18	1.65	1.73	1.66	2.12	1.13	2.88	1.45	0.88	**1.70**	0.84–20.5	0.05–0.22	0.79
Th/Co	35.4	9.12	45.1	30.5	0.16	**24.1**	5.57	12.1	6.63	3.03	4.98	1.76	4.5	11.5	9.65	4.89	**6.46**	0.67–19.4	0.04–1.40	0.63
Th/Cr	3.54	1.21	1.8	3.05	0.36	1.99	0.56	1.09	0.33	0.3	0.99	0.53	0.22	0.57	0.58	0.49	0.56	0.13–2.7	0.018–0.05	0.13
Eu/Eu∗	0.73	0.73	0.72	0.83	1	**0.80**	0.72	0.76	0.77	0.74	0.79	0.74	0.72	0.76	0.73	0.85	**0.76**	0.40–0.94	0.71–0.95	0.65
La/Cr	11.1	6.48	17.2	4.78	3.28	**8.56**	9.92	4.62	2.3	3.76	8.42	5.9	3.88	4.76	11.9	4.03	**5.94**	**-**	–	0.36
Y/Ni	28.3	12.8	56.3	82.8	3.56	**36.7**	6.6	9.35	6.34	5.85	8.67	15.4	12	17.4	17.5	9.58	**10.8**	**-**	–	0.5
Cr/V	0.15	0.33	0.26	0.32	0.03	**0.218**	0.83	0.63	1.33	1.05	0.37	0.41	0.62	1.17	0.83	0.44	**0.76**	**-**	–	0.78
Cr/Th	0.28	0.82	0.55	0.33	2.77	**0.95**	1.79	0.92	3.02	3.31	1.0	1.88	4.4	1.73	1.73	2.04	**2.18**	–	–	7.76
Co/Th	0.02	0.11	0.02	0.03	7.22	**1.48**	0.18	0.08	0.15	0.33	0.2	0.56	0.22	0.08	0.1	0.2	**0.21**	**-**	–	17
Rb/Sr	0.19	0.39	0.56	0.71	0.18	**0.41**	3.97	11.7	6.23	0.68	2.85	0.78	2.7	1.29	2.86	0.79	**3.38**	**-**	–	0.32
(La/Lu)N	5.06	7.78	12.1	1.27	8.18	**6.87**	23.1	5.83	10.1	18.8	11.3	11.5	19.3	9.03	19.6	15.4	**14.4**	**-**	–	10.35
LaN/YbN	4.91	7.29	11.7	1.25	7.52	**6.53**	22.2	5.47	8.91	17.3	11.1	10.8	18.9	8.09	18.9	14.6	**13.6**	**-**	–	10.69
LaN/SmN	2.77	4.31	4.56	2.33	2.10	**3.21**	3.91	2.49	2.81	3.93	3.73	3.34	3.84	3.42	4.05	2.75	**3.43**	**-**	–	4.15
LREE/HREE	4.06	9.25	10.2	1.56	6.11	**6.22**	15.5	6.07	10.4	10.6	9.62	9.66	11.8	6.46	9.67	10.2	**10.0**	**-**	–	8.68
∑REE	459	955	1346	279	194	**647**	471	273	291	643	335	592	381	391	448	469	**429.8**	**-**	–	148

Eu/Eu∗ = EuN/(SmN x GdN)0.5, where N subscript indicates normalization to chondrite.

∑LREE = La + Ce + Pr + Nd + Sm + Eu, and ∑HREE=Gd + Tb + Dy + Ho + Er + Tm + Yb + Lu.

UCC: Upper Continental Crust (Taylor and McLennan, 1985).

## 5 Discussion

### 5.1 Classification of sandstone

Before examining the lithotectonic setting, paleoweathering–paleoclimate, sediment maturity, and depositional environment of the Angot–Gazo intertrappean sediments, geochemical and mineralogical classifications have been verified. These classifications are crucial for further dialog in aforementioned studies, as diverse sedimentary processes result in varied sandstone compositions. For instance, the formation of quartz arenite is typically the result of extended weathering and transport, while the production of lithic and arkose arenite largely depends on the source rock [14]. Alternatively, the process of diagenesis significantly influences the formation of graywacke matrix [15].

**Fig. 4:**
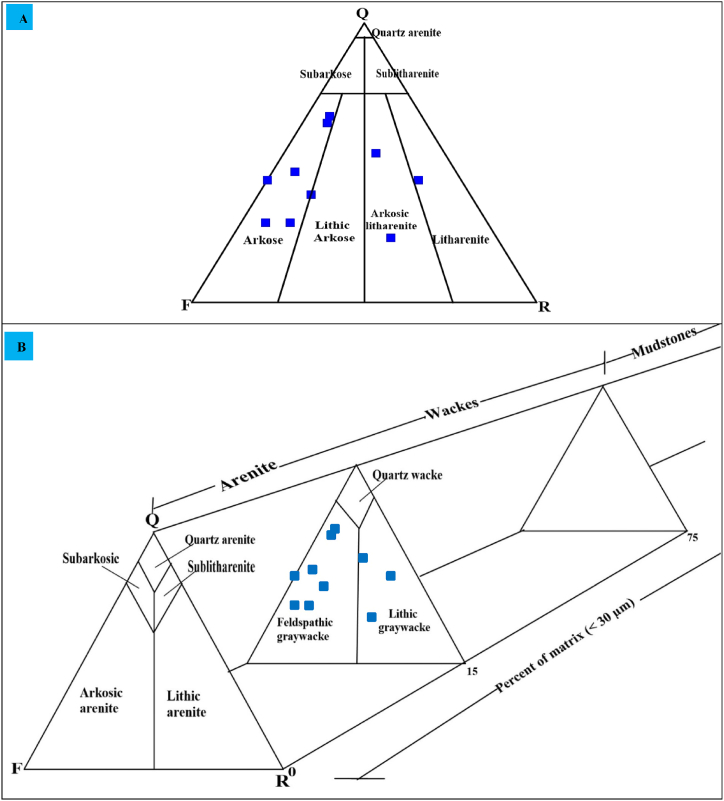
Mineralogical classification of Angot–Gazo sandstone: (A) Folk (1980) sandstone classification based solely on framework mineralogy (Q–Quartz, F–Feldspar and R–Rock fragment), and (B) Pettijohn (1972) sandstone classification based on both framework mineralogy and groundmass.

**Fig. 5:**
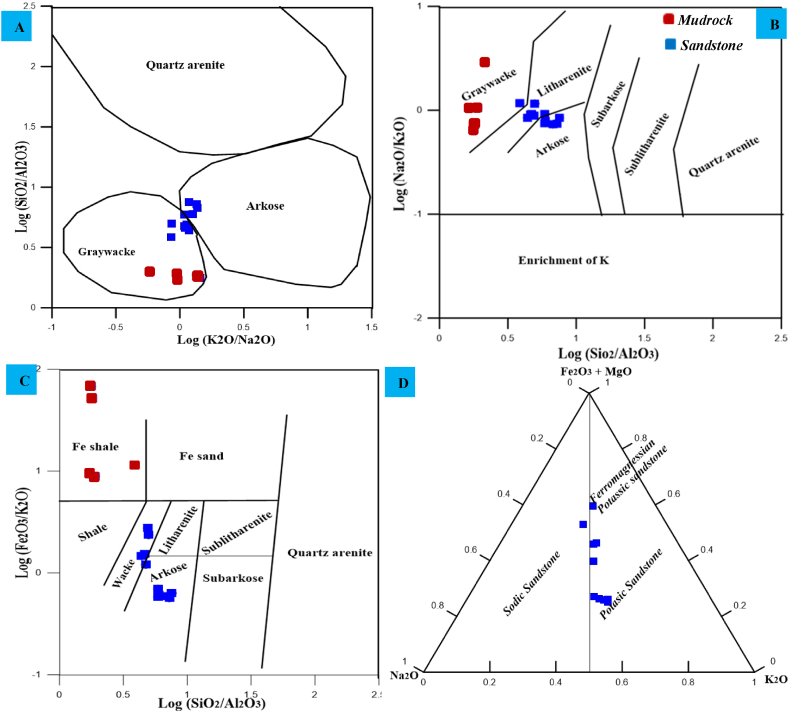
Geochemical classification of Angot–Gazo intertrappean sediments: (A & B) Log (K_2_O/Na_2_O) vs. Log (SiO_2_/Al_2_O_3_), and Log (SiO_2_/Al_2_O_3_) vs. Log (Na_2_O/K_2_O) classification after Pettijohn et al. (1972), (C) Log (SiO_2_/Al_2_O_3_) vs. Log (Fe_2_O_3_/K_2_O) diagram of Herron (1988), and (D) trinary diagram of Na_2_O–(MgO + Fe_2_O_3_)–K_2_O classification after Crook et al. (1974).

The present study developed mineralogical and geochemical classification diagrams to classify the Angot–Gazo intertrappean sandstone. The QFR (quartz, feldspars, and rock fragments) classification method was primarily used for sandstone mineralogical classifications. Both the Folk and Pettijohn sandstone classification systems were employed for this study, which are based on recalculated framework composition. The Folk categorization system relies on three framework grains (Q–F–R), but exclude the groundmass (Fig. 4A). Consequently, the examined terrestrial sandstone samples are categorized within the range from arkose to litharenite type, as indicated by this mineralogical plot. Most samples (six of them) fall into the arkose category, two samples are classified as arkosic litharenite, and the remaining two samples are categorized as lithic arkose and litharenite types. The subsequent categorization scheme, developed by Dott and Pettijohn, is more expressive than the previous one, as it is primarily considers the relative abundance of matrix and framework minerals [16,17]. Sandstones with minimal matrix content (<15 %) are categorized as arenite (lithic, arkosic, or quartz) based on the proportionate abundance of framework ingredients. In contrast, sandstones with a matrix content greater than 15 % are termed greywacke (lithic, arkosic, or quartz). The Angot–Gazo sandstone, which contains a matrix in the range of 20–35 % with an average of 27 % (Table 1), is therefore classified in the arkosic and lithic greywacke fields (Fig. 4B). Several geochemical diagrams based on major oxides and trace elements, as proposed by Refs. [[18], [19], [20]], are used to characterize clastic sedimentary rocks. According to the classification by Ref. [18], the examined samples are categorized as arkose, litharenite, and greywacke (Fig. 5A and B). In comparison, all mudrock samples are plotted under graywacke fields. Similarly, the variation diagram from Ref. [20] demonstrates that each sample is plotted in the fields of wacke, litharenite, and arkose (Fig. 5C). However, all mudrock samples are plotted on iron shale fields. This is correlated with field observation of mudrock, which is consistent with field observations indicating that the mudrock has a reddish color due to iron content. The first three variation diagrams suggest that Angot–Gazo terrestrial sandstone is immature, with all samples falling in the fields of arkose, litharenite, and graywacke. The final ternary diagram, Na_2_O–(MgO + Fe_2_O_3_)–K_2_O, is also helpful for further classifying the sandstone into potassic and sodic type [19]. Accordingly, most of the samples (eight of them) fall into the potassic sandstone category, while the other two samples fall into sodic and ferromagnesian potasic sandstone fields (Fig. 5D). This geochemical plot is consistent with the mineralogical observations, both of which show an enrichment of alkaline feldspar over plagioclase. Overall, both the mineralogical and geochemical variation diagrams suggest that the Angot–Gazo plateau contains immature sandstone, which is predominantly arkosic greywacke.

### 5.2 Provenance

The presence of several characteristics that have been noted in the field helps to identifying the provenance of intertrappean sediments. The sandstone observed is massive, medium- to coarse-grained, sub-rounded to angular grains, and exhibits poor to moderate sorting. It is primarily composed of feldspar and quartz minerals, with minor rock fragments. Some quartz and feldspar minerals in hand specimens display their original crystal habits–hexagonal and tabular, respectively. The rock fragments vary in size, ranging from a few millimeters to 10 cm in diameter (Fig. 2G). These lithic fragments include volcanic glass, rhyolite, and ignimbrite. The framework grains of the sandstone are cemented by ash material ranging in color from brownish-gray to whitish. Accordingly, the early-formed felsic pyroclastic rocks were likely the primary source rocks. As the microscopic study shows, the sandstone is composed of quartz, feldspar, lithic fragments, ash material as groundmass, and some opaque minerals. Notably, no mafic minerals are present in the sandstone. Even the feldspar minerals are predominantly sanidine and orthoclase, with only a few plagioclases. This suggests that the primary source materials for the Angot–Gazo intertrappean sediments were felsic rocks.

Discrimination function (DF) analysis is an essential tool for tracing the origin of sandstone [21]. It is considered to distinguish between four sedimentary provenance fields: felsic, intermediate, mafic igneous, and quartzose recycled sedimentary. The following equations, (eq 1), (eq 2), are utilized to calculate the discriminant functions based on the main oxide composition:

(eq 1) **Discriminant function1** = 30.638TiO_2_/Al_2_O_3_ – 12.541Fe_2_O_3_/Al_2_O_3_ + 7.329MgO/Al_2_O_3_ + 2.031Na_2_O/Al_2_O_3_ + 35.402K_2_O/Al_2_O_3_ – 6.382

(eq 2) **Discriminant function2** = 56.500TiO_2_/Al_2_O_3_ – 10879Fe_2_O_3_T/Al_2_O_3_ + 30.875MgO/Al_2_O_3_ – 5.404Na_2_O/Al_2_O_3_ + 11.112K_2_O/Al_2_O_3_ – 3.89

**Fig. 6:**
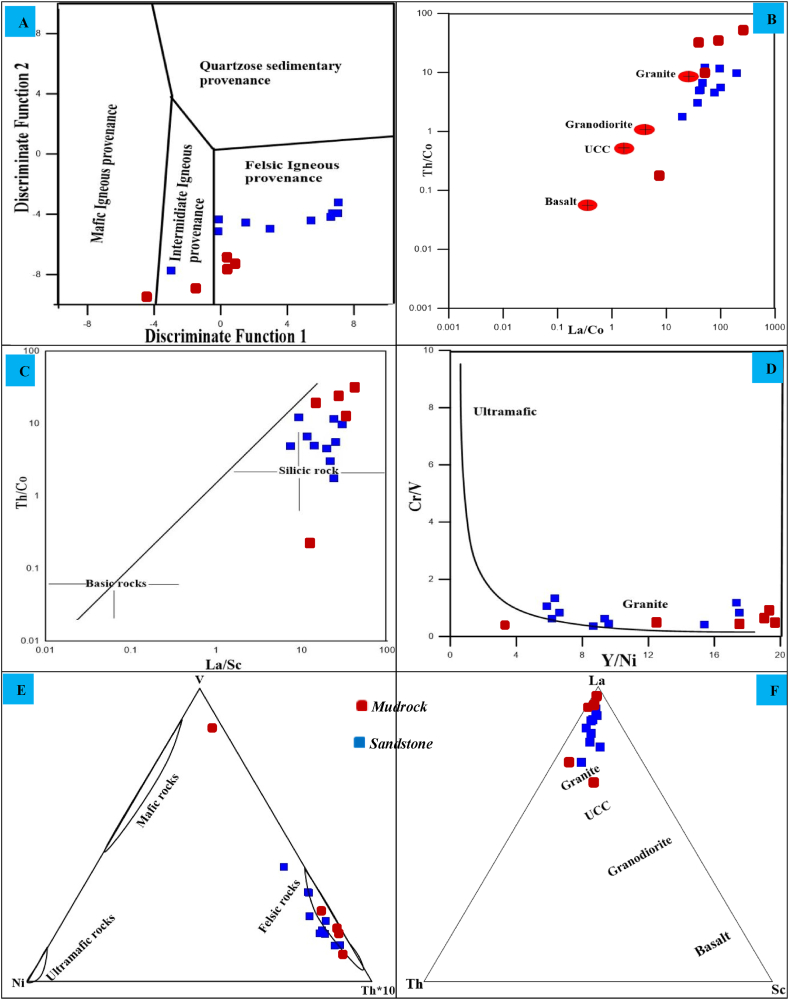
Provenance signatures for Angot–Gazo intertrappean sediments: (A) Discriminant function diagram after Roser and Korsch, 1988, (B–D) binary plots based on La/Co–Th/Co ratio (after Cullers and Berendsen 1998), La/Sc–Th/Co ratio (after Cullers, 2000), and Y/Ni–Cr/V ratio (after Mongelli et al., 2006), and (E–F) ternary plot based on V–Ni–Th∗10 (after Jahn and Condie, 1995) and La–Th–Sc (after Bracciali et al., 2007).

Most of the Angot–Gazo sandstone samples (nine out of ten) plot within the felsic igneous provenance, with one sample falling into the intermediate igneous provenance field (Fig. 6A). Mudrock samples also exhibit similar features, except for one sample, which falls in the mafic region. The Al_2_O_3_/TiO_2_ ratio is also vital for determining the provenance of terrigenous sedimentary rocks. This ratio typically ranges from 70 to 21 in felsic igneous rocks, 21–8 in intermediate rocks, and 8–3 in mafic igneous rocks [22]. The Al_2_O_3_/TiO_2_ ratio values for the Angot–Gazo sandstone range from 15.3 to 25.29 %, indicating that the parent material is likely intermediate to felsic igneous rock.

By interpreting high field strength elements (Zr, Hf, Y, Ta, and Nb), transition elements (Sc, Co, Cr, and Ni), and rare earth elements (REEs) in conjunction with Al, Ti, and Th concentration can be used to discriminate the provenance composition of clastic sedimentary rocks [[23], [24], [25]]. These elements reflect the signature of the parent materials, as they are quantitatively transferred into clastic sediments during the process of sedimentation. For this reason, this study employs five trace element-based discrimination plots to know the provenance of the sediments. In the bivariate plots of La/Co–Th/Co, La/Sc–Th/Co, and Y/Ni–Cr/V, all analyzed samples reflect a felsic source (Fig. 6B–D). The binary plot of La/Sc–Th/Co ratios (Fig. 6C) suggests that the samples are derived from silicic rocks. Sediments from ultramafic to mafic sources typically exhibit low Y/Ni ratio (<1) and high Cr/V ratios (substantially >1) [26,27]. In contrast, the Angot–Gazo mudrock and sandstone samples have high Y/Ni ratios (avg. 36.7 and 10.8, respectively) and very low Cr/V ratios (avg. 0.22 and 0.76, respectively), which indicate a typically felsic source. Graphically, all samples lie within the felsic igneous provenance field (Fig. 6D). Likewise, all analyzed samples in the triangular plot of V–Ni–Th∗10 and La–Th–Sc (Fig. 6E and F) suggest derivation from felsic rocks, with both plots showing a higher enrichment of thorium and lanthanum compared to their respective elemental counterparts.

**Fig. 7:**
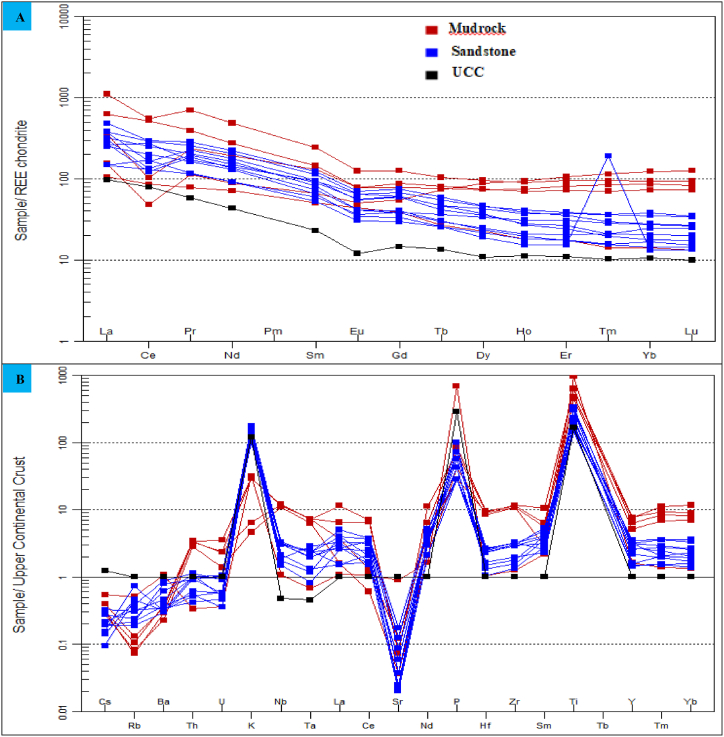
REE patterns and multi-element plots for Angot–Gazo intertrappean sediments: (A) Chondrite normalized REE pattern (after Boynton, 1984), and (B) multi-element spider plot (after Taylor and McLennan, 1995) for Angot–Gazo intertrappean sediments. UCC data is from Taylor and McLennan (1985).

According to Ref. [25], sediments derived from felsic rocks have high LREE/HREE ratios, fractionated patterns, and negative Eu anomalies. Conversely, sediments derived from mafic rocks display lower LREE/HREE ratios, minimal or no Eu anomalies, and less fractionated patterns. The Angot–Gazo terrestrial sediments chondrite–normalized REE pattern illustrate a slight depletion in HREEs and significant enrichment in LREEs (Fig. 7A). The studied mudrock and sandstone samples show fractionated and nearly smooth REE patterns, with slight negative Eu anomaly (averaging, 0.80 & 0.76, respectively). The observed REE patterns and high LREE/HREE ratios for both mudrock (ranging from 1.56 to 10.2; avg. 6.22) and sandstone (ranging from 6.07 to 15.5; avg. 10.0) implies that these sediments are resulted from felsic bedrock (Shao et al., 2001). Additionally, high field strength elements like Zr, Hf, Y, Ta, and Nb, along with REEs such as La, Nd, Tm, Yb, Ce, and Y, are significantly more abundant in the investigated sediments than in the upper continental crust (UCC) (Fig. 7B). These elemental enrichments further indicate that the sediments are drived from felsic and fractionated sources.

Other elemental ratios such as Al/Ti, La/Sc, La/Co, Th/Sc, Th/Co, Th/Cr, and Eu/Eu∗ were taken into consideration in order to more precisely define the felsic versus mafic origins of the Angot–Gazo sediments. These elements retain the properties of their source and remain immobile when they exposed to the surface [25]. The elemental ratios of the Angot–Gazo mudrock and sandstone fall within the range characteristics of felsic sources, indicating that felsic rocks are the principal source material (Table 4). Felsic igneous rocks and their weathering products often exhibit greater abundances of elements like La, Th, U, and Y, while mafic rocks contain higher concentrations of Co, Sc, Ni, and Cr [28]. Consequently, the Th/Sc, Th/Cr, Th/Co, La/Sc, La/Cr, and Y/Ni ratios of the Angot–Gazo intertrappean sediments are noticeably elevated (Table 4). In general, these ratio values, combined with the results from the discrimination plots, consistently suggest a felsic source composition.

### 5.3 Tectonic setting

**Fig. 8:**
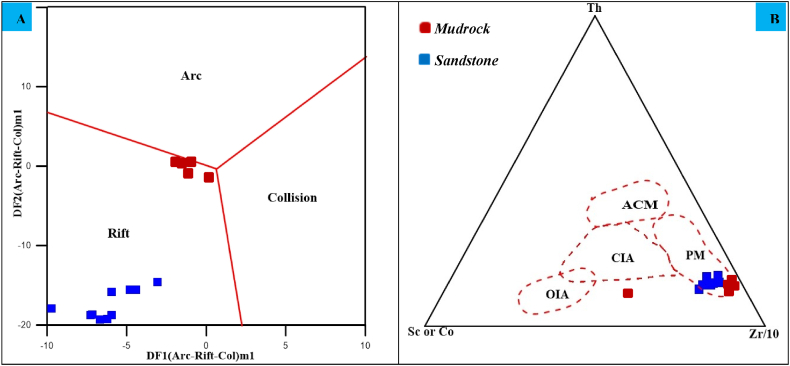
Tectonic setting discrimination plots for the studied sediments, (A) DF1 (arc–rift–col)m1 vs. DF2 (arc–rift–col)m1 after Verma et al. (2013), (B) Zr/10–Th–Sc or Co (after Bhatia and Cook, 1986). Dashed lines represent various tectonic settings (OIA- Oceanic Island Arc; CIA- Continental Island Arc; ACM- Active Continental Margin and PM- Passive Continental Margin).

Many writers have employed the geochemistry of clastic sedimentary rocks, namely immobile elements, to specify the tectonic settings [21,29]. To understand the tectonic environment of the Angot–Gazo terrestrial deposits, this study used two discrimination diagrams. The first one is a new discriminant function multi-dimensional plot, constructed based on major oxide composition so as to differentiate three tectonic settings: arc, continental rift, and collision [30,31]. Equations (eq1a), (eq2a) were utilized to calculate the discriminant function for sandstone, where the silica concentration is high (SiO_2_)adj >63 %. On the other hand, equations (eq3), (eq4) were utilized to calculate the discriminant function results of mudrock, because of its low silica content, (SiO_2_)adj ≤63 %. The (SiO_2_)adj talks about the measured silica value after volatile-free adjustment of the ten major elements to 100 wt % [30]. Accordingly, both the studied mudrock and sandstone samples are plotted within the continental rift region (Fig. 8A).

(eq1a) DF1(Arc-Rift-Col)M1 = (−0.263 × ln(TiO_2_∕SiO_2_)adj) + (0.604 × ln(Al_2_O_3_∕SiO_2_)adj) + (−1.725 × ln (Fe_2_O_3_∕ SiO_2_)adj) + (0.660 × ln(MnO∕ SiO_2_)adj) + (2.191 × ln(MgO∕SiO_2_)adj) + (0.144 × ln(CaO∕SiO_2_)adj) + (−1.304 × ln(Na_2_O∕SiO_2_)adj) +(0.054 × ln(K_2_O∕SiO_2_)adj) +(-0.330 × ln (P_2_O_5_∕SiO_2_)adj) + 1.588

(eq2a) DF2(Arc-Rift-Col)M1 = (1.196 × ln(TiO_2_∕SiO_2_)adj) + (1.064 × ln(Al_2_O_3_∕SiO_2_)adj) + (0.303 × ln(Fe_2_O_3_∕ SiO_2_)adj) +(0.436 × ln(MnO∕SiO_2_)adj) + (0.838 × ln(MgO∕SiO_2_)adj) + (0.407 × ln(CaO∕SiO_2_)adj) + (1.021 × ln(Na_2_O∕SiO_2_)adj) + (−1.706 × ln(K_2_O∕SiO_2_)adj) + (−0.126 × ln(P_2_O_5_∕SiO_2_)adj) −1.068

(eq3) DF1(Arc-Rift-Col)M2 = (0.608 × ln(TiO_2_∕SiO_2_)adj) + (−1.854 × ln(Al_2_O_3_∕SiO_2_)adj) + (0.299 × ln(Fe_2_O_3_∕ SiO_2_)adj) + (−0.550 × ln(MnO∕SiO_2_)adj) + (0.120 × ln(MgO∕SiO_2_)adj) + (0.194 × ln(CaO∕SiO_2_)adj) + (−1.510 × ln(Na_2_O∕SiO_2_)adj) + (1.941 × ln(K_2_O∕SiO_2_)adj) + (0.003 × ln (P_2_O_5_∕SiO_2_)adj) – 0.294

(eq4) DF2(Arc-Rift-Col)M2 = (−0.554 × ln(TiO_2_∕SiO_2_)adj) + (−0.995 × ln(Al_2_O_3_∕SiO_2_)adj) + (1.765 × ln(Fe_2_O_3_∕ SiO_2_)adj) + (−1.391 × ln(MnO∕SiO_2_)adj) + (−1.034 × ln(MgO∕SiO_2_)adj) + (0.225 × ln(CaO∕SiO_2_)adj) + (0.713 × ln(Na_2_O∕SiO_2_)adj) + (0.330 × ln(K_2_O∕SiO_2_)adj) + (0.637 × ln(P_2_O_5_∕SiO_2_)adj) −3:631

Where, DF implies discriminant function, Col = collision, M1 = high-silica and M2 = low silica.

The second tectonic discrimination ternary diagram utilizes trace components such as Zr/10–Th–Sc or Co [32]. This diagram distinguishes between four distinct tectonic setting fields: ACM (Active Continental Margin), PM (Passive Continental Margin), OIA (Oceanic Island Arc), and CIA (Continental Island Arc). Consequently, all the sediment samples under study are plotted within the passive continental margin fields (Fig. 8B). However, one mudrock sample falls outside all the defined tectonic setting regions.

### 5.4 Paleoweathering and paleoclimate

The strength of weathering is primarily determined by the intricate interplay of tectonic setting (including relief: slope angle, and elevation differences), vegetation, climate, and the relative vulnerability of parent rocks to weathering [33]. Analyzing the mineralogy, texture, and geochemistry of sediments is essential for understanding weathering intensity [[34], [35], [36]]**.** The presence of angular to sub-rounded mineral grains, variably sized rock fragments, and the poorly sorted nature of the sandstone, combined with the significant occurrence of feldspar minerals, indicates limited weathering and short transport distances for Angot–Gazo intertrappean sediments, as evidenced both in hand specimens and under transmitted light microscopy (Fig. 2, Fig. 3). Additionally, typical sandstone structures like ripple marks, horizontal bedding, and cross-bedding are absent in the studied sandstone. These observations suggest that the source rocks likely originated in high-relief areas that experience fast erosion with minimal weathering.

The extent of past weathering and its principal paleoclimatic conditions can be determined using several weathering indices: the Chemical Index of Alteration (CIA), Chemical Index of Weathering (CIW), Plagioclase Index of Alteration (PIA), and Mineralogical Index of Alteration (MIA), as proposed by Refs. [[37], [38], [39], [40]]. These indices are calculated as follows: CIA = (Al₂O₃/(Al₂O₃+CaO∗+Na₂O + K₂O)) × 100, CIW = (Al₂O₃/(Al₂O₃+CaO∗+Na₂O)) × 100, PIA = [(Al₂O₃–K₂O)/(Al₂O₃+CaO∗+Na₂O–K₂O)] × 100 and MIA = 2 × (CIA–50) (where CaO∗ represents the amount of CaO present solely in the silicate fraction).

The Angot–Gazo sandstone exhibits low to moderate CIA, CIW, and PIA values, while the mudrock shows higher values (Table 2). CIA values for the sandstone range from 58.22 % to 70 %, with an average of 64.06 %, whereas the mudrock ranges from 80.22 % to 98.88 %, averaging 92.55 %. These lower to intermediate CIA values in the sandstone suggest slight to moderate weathering under arid to semi-arid conditions, whereas the mudrock indicates deep weathering predating the sandstone [37,39]. Weathering effects are further evaluated using the Chemical Index of Weathering [38]. CIW values for the studied sandstone range from 75.75 % to 82.9 %, with an average of 78.72 %, and for the mudrock, from 85.28 % to 99.34 %, with an average of 94.92 %. These results indicate that the source rocks experienced moderate to high weathering in the sandstone and mudrock profiles, respectively.

The chemical weathering of parent rocks can be further assessed using the Plagioclase Index of Alteration (PIA) and the Mineralogical Index of Alteration (MIA). The Angot–Gazo sandstone samples exhibit PIA values ranging from 65.35 to 79.06 (avg. 71.9), and mudrock samples range from 84.3 to 99.3 (avg. 94.67). These values indicate that plagioclase in the source area underwent moderate to intense weathering in the sandstone and mudrock formations, respectively. The Mineralogical Index of Alteration (MIA) categorizes weathering as follows: 0–20 % incipient, 20–40 % weak, 40–60 % moderate, and 60–100 % extreme [40]. The MIA values for the investigated sandstone range from 16.4 to 40 %, with an average of 28.12 %, indicating weak weathering, whereas the mudrock ranges from 60.45 to 97.76 %, with an average of 85.11 %, suggesting intense weathering.

**Fig. 9:**
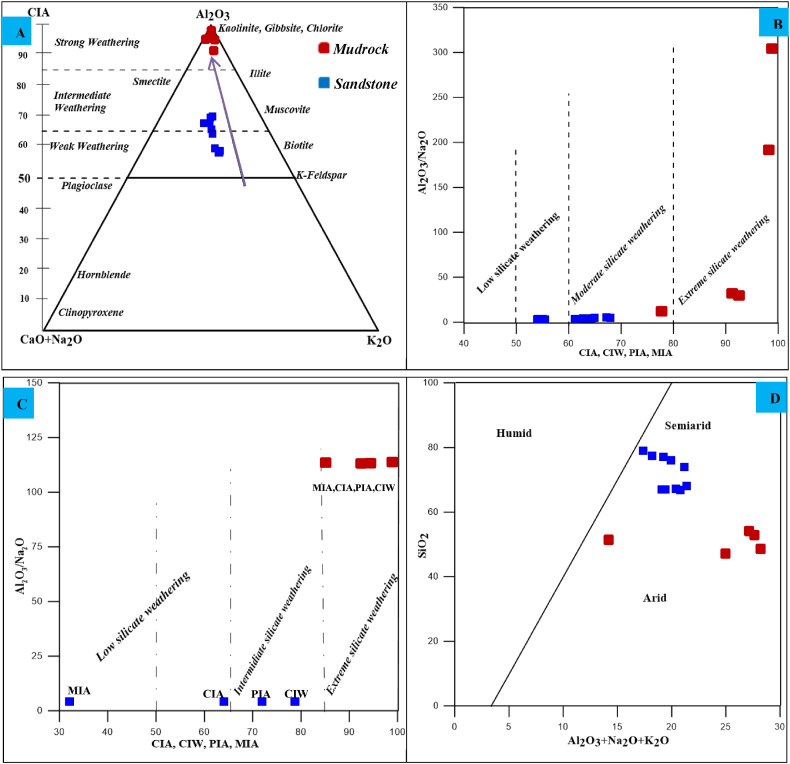
Weathering and paleoclimatic condition plots for Angot–Gazo intertrappean sediments: (A) A-CN-K ternary diagram (after Nesbitt and Young, 1984) with a CIA scale, (B) a binary plot of Al₂O₃/Na₂O vs. average values of various chemical weathering indices (CIA, CIW, PIA, & MIA), (C) a binary plot of Al₂O₃/Na₂O vs. individual average values of CIA, CIW, PIA, & MIA (after Kandasamy and Chen-Tung Chen, 2006), and (D) a binary diagram SiO_2_ vs. Al_2_O_3_+K_2_O + Na_2_O illustrating the paleoclimatic conditions of Angot–Gazo terrestrial sediments (after Suttner and Dutta, 1986).

Nesbett et al. (1996) utilized the A-CN-K ternary diagram (Al₂O₃–(CaO + Na₂O)–K₂O) to deduce weathering trends [37,41]. The A-CN-K diagram for the Angot–Gazo sandstone displays a slight to intermediate degree of weathering, while the mudrock shows a strong weathering history (Fig. 9A). In this diagram, the sandstone samples plot near their supposed parent rock around the center, while the highly altered mudrock samples plot at the peak of Al₂O₃ end. In addition, the binary scheme of Al₂O₃/Na₂O against average values of various chemical weathering indices (CIA, CIW, PIA, and MIA), along with a binary diagram of Al₂O₃/Na₂O versus individual average values of these indices, illustrates a slight to moderate degree of weathering for the sandstone and strong weathering for the studied mudrock (Fig. 9B and C). Paleoclimatic circumstances also significantly determining the sediment maturity by influencing the rate of weathering and sediment movement [42]. For instance, arid and semi-arid climates commonly result immature sediments because of negligible chemical modification. The major oxide-based binary diagram (SiO_2_ vs. Al_2_O_3_+K_2_O + Na_2_O) indicated by Ref. [43] is helpful for illustrating the paleoclimatic conditions of clastic sedimentary rocks. According to this diagram, the Angot–Gazo terrestrial sediment samples fall between semi-arid and arid domains (Fig. 9D).

### 5.5 Sediment maturity and recycling

Extensive weathering results mature sediments, typically found in humid, gently sloped, and hot environments, while minimal weathering creates immature sediments, mostly in steep slope, dry, and cold conditions [33]. The textural and compositional maturity of the Angot–Gazo sandstone is determined through hand specimens, petrography, and geochemical analysis. Compositional maturity refers to the proportion of unstable versus stable grains in the sandstone, while textural maturity is defined by the sorting, rounding of mineral grains, and the abundance of the matrix [33]. The summarized value of feldspar, rock fragments, and ash materials are predominate over quartz grains, indicating the compositional immaturity of the Angot–Gazo sandstone (Table 4). Consistently, the grains of the studied sandstone are predominantly angular to sub-rounded, poorly to moderately sorted, and ash-rich, indicating textural immaturity (Fig. 3A–L). However, a few quartz grains show well-rounded features under petrographic analysis (Fig. 3A, B & G). These quartz grains likely underwent sediment recycling, beginning with the mudrock formation. In the early stage of terrestrial sedimentation, intense weathering (as evidenced by CIA, CIW, PIA, & MIA indices) resulted in clay to silt-sized mudrock at the base of the Angot–Gazo intertrappean sediments. Additionally, some quartz grains might have transported over moderate distances on gentle slopes. Except for a few quartz grains, most framework grains exhibit angular to sub-angular shapes, reflecting both immaturity and initial sediment recycling.

**Fig. 10:**
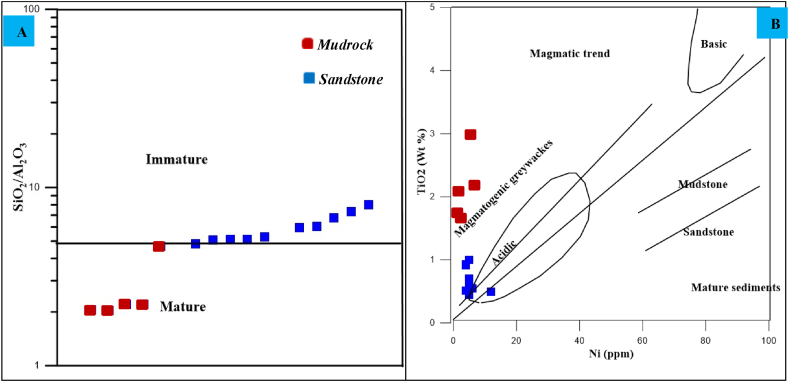
Maturity diagrams for Angot–Gazo intertrappean sediments: (A–B) Binary diagrams based on SiO_2_/Al_2_O_3_ ratio and Ni–TiO_2_ value (after Floyd et al., 1989).

This study also examined the chemical makeup of the sandstone to determine its maturity. A relatively low ratio of immobile to mobile components is a characteristic of young sediments [39,42,44]. Typically, a high SiO₂/Al₂O₃ ratio (>10) and elevated SiO₂ content indicate chemical maturity and a quartz-rich nature [45,46]. However, the Angot–Gazo intertrappean sandstone is characterized by a low SiO_2_/Al_2_O_3_ ratio (ranging from 4.4 to 7.56, avg. 5.7 wt%) and a high SiO₂ content (avg. 71.9 wt%). This lesser SiO₂/Al₂O₃ ratio reflects a significant clay content, highlighting the sandstone's chemical immaturity. Furthermore, the SiO₂/Al₂O₃ ratio and Ni–TiO_2_ based maturity discrimination diagram were utilized to differentiate between immature and mature sediments [47]. The analyzed samples were classified as immature, formed from a magmatic source (Fig. 10A and B). Thus, the investigated sandstone's immaturity is evident not only in its textural and mineralogical characteristics but also in its chemical composition.

### 5.6 Depositional enviroment

The depositional environment of the Angot–Gazo intertrappean sediments can be inferred from the existing sedimentary unit sequences and the ratio values of different trace elements. The studied terrestrial sediments are sandwiched by felsic volcanic rocks, with a sequence beginning with massive mudrock at the base, representing deposition in a water pond sub-basin or lake water environment. This is followed by massive sandstone, which represents debris inflow transported over a short distance by rivers. The massive and immature nature of these terrestrial sediments represent rapid sedimentation, characterized by short-lived weathering, erosion, and deposition under semi-arid climatic conditions (Fig. 9D).

**Fig. 11:**
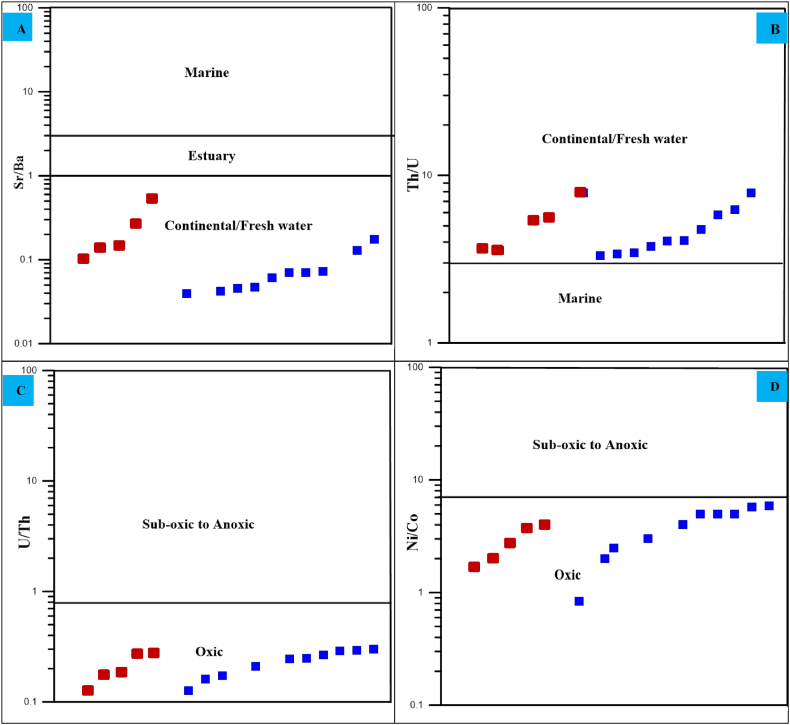
Depositional environment and paleo-oxidation condition discrimination diagrams for Angot–Gazo intertrappean sediments: (A–B) based on Sr/Ba and Th/U ratios, and (C–D) using U/Th and Ni/Co ratios (after Bokanda et al., 2019).

In addition, various trace element ratios were employed by the authors to ascertain the depositional settings of sedimentary rocks. According to Wang 2021, the Sr/Ba ratio value of sediments can reflect the depositional environment: values > 8 indicate a marine environment, 3–8 indicate a pro-delta (salt) environment, 1–3 indicate a delta front (brackish) environment, and values < 1 indicate a freshwater environment [48]. The average Sr/Ba ratio for all mudrock and sandstone samples under study is extremely low, with value < 1 (avg. 0.24 and 0.07, respectively). These low ratio values, along with their corresponding plots, show a freshwater (continental) environment (Fig. 11A). Whorlow (1993) notes that the Th/U ratios of <2 indicate a marine environment, while ratios ≥2 suggest a continental setting, as cited in Ref. [49]. Accordingly, the Th/U ratios for the studied mudrock and sandstone units are >2 (averaging 5.2 and 4.67, respectively), further supporting a continental depositional environment (Fig. 11B). The Ni/Co and U/Th ratios are also significant markers for the paleo-oxidation conditions of sedimentary rocks [50,51]. Sedimentary rocks with U/Th ratios ≤0.75 indicate oxygenated circumstances, while ratios between 0.75 and 1.25 often infer dioxic to anoxic environments 0.75–1.25 often indicate dioxic to anoxic environments [50]. The studied terrestrial sediments (both mudrock and sandstone) have U/Th ratios <0.75, placing them within an oxic depositional setting (Fig. 11C). Likewise, Ni/Co ratios ≤5 show oxic environments; whilst ratios of 5–9 indicate dysoxic to anoxic conditions [51,52]. Most examined mudrock and sandstone samples have Ni/Co ratio values less than five (avg. 2.57 and 4.06, respectively), representing an oxic depositional domain (Fig. 11D).

## 6 Conclusion

The Angot–Gazo intertrappean sedimentary rocks were studied through comprehensive field mapping, petrographic and geochemical analysis, yielding the following key findings:

1. The intertrappean sediments (mudrock and sandstone) are principally surrounded by volcanic glass in the upper portion and ignimbrite in the lower half. The sandstone is characterized by medium- to coarse-grained, angular to sub-rounded, and poorly sorted features in hand specimens. Petrographic study reveals the existence of rock fragments, feldspar, and quartz as framework grains, with proportional amounts of ash material as the matrix.
2. Both mineralogical (based on Q-F-R-ground mass) and geochemical classification plots (based on Log (K_2_O/Na_2_O) vs. Log (SiO_2_/Al_2_O_3_), Log (SiO_2_/Al_2_O_3_) vs. Log (Na_2_O/K_2_O), Log (SiO_2_/Al_2_O_3_) vs. Log (Fe_2_O_3_/K_2_O), and Na_2_O-(MgO + Fe_2_O_3_)-K_2_O) essentially categorize the studied sandstone as arkosic to lithic greywacke.
3. Multiple provenance discrimination diagrams, Chondrite-normalized REE patterns, and elemental ratios (e.g., La/Cr, La/Sc, La/Co, Th/Sc, Th/Cr, Th/Co, Y/Ni, LREE/HREE & Eu/Eu∗) imply that the examined sediments result from the weathering of felsic volcanic rocks.
4. The tectonic setting discrimination plots generated from DF1& 2 (arc–rift–col)m1, and the Zr/10–Th–Sc or Co ternary diagram revealed a rift tectonic setting and a passive continental margin, respectively.
5. The A-CN-K ternary diagram, weathering parameters such as CIA, CIW, PIA, and MIA in conjunction with SiO_2_ vs. Al_2_O_3_+K_2_O + Na_2_O plot, illustrates slight to moderate, and strong weathering in the source area for the studied sandstone and mudrock, respectively, under semi-arid paleoclimatic conditions.
6. Based on textural, mineralogical, and geochemical data interpretation (SiO_2_/Al_2_O_3_ and Ni–TiO_2_ plot), the studied sandstone is deemed immature.
7. The depositional environments of the Angot–Gazo sediments were studied using U/Th, Ni/Co, Sr/Ba, and Th/U ratio plots, which demonstrate oxic depositional conditions within fresh or lacustrine water.

## Data availability statement

All laboratory data are presented in the main body in table format. Any additional data will be made available upon request.

## CRediT authorship contribution statement

**Adise Zemelak:** Writing – review & editing, Writing – original draft, Visualization, Validation, Supervision, Software, Resources, Project administration, Methodology, Investigation, Funding acquisition, Formal analysis, Data curation, Conceptualization. **Worash Getaneh:** Supervision, Resources, Investigation, Conceptualization. **Dereje Ayalew:** Visualization, Resources, Methodology. **Dejen Teka:** Supervision, Methodology, Investigation, Formal analysis. **Abebaw Bitew:** Writing – original draft, Methodology, Investigation, Formal analysis, Data curation. **Asaye Getenet:** Validation, Software, Investigation.

## Declaration of competing interest

The authors declare that they have no known competing financial interests or personal relationships that could have appeared to influence the work reported in this paper.
